# Credit Risk Modeling Using Transfer Learning and Domain Adaptation

**DOI:** 10.3389/frai.2022.868232

**Published:** 2022-05-03

**Authors:** Hendra Suryanto, Ashesh Mahidadia, Michael Bain, Charles Guan, Ada Guan

**Affiliations:** ^1^Rich Data Corporation, Sydney, NSW, Australia; ^2^School of Computer Science and Engineering, The University of New South Wales, Sydney, NSW, Australia

**Keywords:** credit risk, transfer learning, domain adaptation, explainable AI, deep learning

## Abstract

In the domain of credit risk assessment lenders may have limited or no data on the historical lending outcomes of credit applicants. Typically this disproportionately affects Micro, Small, and Medium Enterprises (MSMEs), for which credit may be restricted or too costly, due to the difficulty of predicting the Probability of Default (PD). However, if data from other related credit risk domains is available *Transfer Learning* may be applied to successfully train models, e.g., from the credit card lending and debt consolidation (CD) domains to predict in the small business lending domain. In this article, we report successful results from an approach using transfer learning to predict the probability of default based on the novel concept of *Progressive Shift Contribution* (PSC) from source to target domain. Toward real-world application by lenders of this approach, we further address two key questions. The first is to explain transfer learning models, and the second is to adjust features when the source and target domains differ. To address the first question, we apply Shapley values to investigate how and why transfer learning improves model accuracy, and also propose and test a domain adaptation approach to address the second. These results show that adaptation improves model accuracy in addition to the improvement from transfer learning. We extend this by proposing and testing a combined strategy of feature selection and adaptation to convert values of source domain features to better approximate values of target domain features. Our approach includes a strategy to choose features for adaptation and an algorithm to adapt the values of these features. In this setting, transfer learning appears to improve model accuracy by increasing the contribution of less predictive features. Although the percentage improvements are small, such improvements in real world lending could be of significant economic importance.

## 1. Introduction

In 2014, 42% of all adults worldwide reported borrowing in the previous year (excluding credit cards). Adults in underdeveloped nations borrow three times as much from family members and friends as from financial institutions. Borrowing through an institution offers advantages over borrowing from family or friends since it provides access to adequate funds and, presumably, better credit conditions under regulation (World Bank, 2017). Access to formal credit has become an issue for young adults in developed countries too. A recent survey by Bankrate found that 58% of millennials (born between 1981 and 1996) in the United States have been denied at least one financial product because of their credit score (BankRate, 2019). Consequently, fintech-based financial products, such as Alipay, Affirm, Klarna, Paypal Credit, and Afterpay have become popular with millennials and Generation Z (born between 1997 and 2010), although with these platforms credit is typically provided in very restricted domains, such as for retail purchases.

Applications for unsecured consumer loans such as credit cards and debt consolidation (CD) loans are common. They are typically scored by algorithms that are mostly based on a person's credit score, income, spending, and other factors such as job and housing stability. This area is now a crowded and competitive marketplace that has been helped by recent fintech activities (especially in the US, UK, and China). These activities have amassed historical data and, consequently, reliable and accurate scoring models. Small company financing is a relatively new sector for fintechs; it is riskier, more diversified, more difficult to forecast outcomes, and lacks data. Although public datasets on this type of lending are scarce (apart from exceptions like the Lending Club data used in this article^1^), it is known that the quantity of historical lending outcomes for small business loans is typically far lower than for other lending types, making it very difficult to develop an accurate and stable model using traditional supervised learning. Since small company lending has less competition and larger margins than consumer lending, finding strategies to forecast loan outcomes and service this market is potentially more profitable for lenders. MSMEs are also one of the most powerful generators of economic development, innovation, and employment. MSMEs typically cite a lack of access to capital as a major stumbling block to expansion. Providing possibilities for MSMEs in developing markets is a critical step toward economic growth and poverty reduction. There are 65 million unmet financial requirements in developing nations (or 40% of formal MSMEs). Forum (2019) estimates that the MSME financing gap in developing nations is $5.2 trillion, which is 1.4 times the present level of MSME lending.

However, resolving these concerns presents various difficulties. When a lender enters new market segments, a new credit risk model is necessary to evaluate loan applicants' credit risk. The current strategy relies on expert rules, in which a credit risk expert develops business rules based on data and their expertise and knowledge. Lenders begin by collecting sufficient labeled data with an expert model in order to develop a supervised learning model. A comparison is made between the expert model and the supervised learning model. The superior model is adopted if one model outperforms the other by a significant margin. In another way, if both models work well together, they can be combined into an “ensemble” model. Lenders in commercial lending systems usually charge more money or limit the amount of credit they can give out because there are not enough labeled data to test the expert models. Consequently, many individuals and small enterprises are shut out of these “conventional” financing systems. When sensitive and personal data can only be accessed on-site by authorized people, it may be hard to get a suitable expert to analyze such data.

Transfer learning is proposed as a connection between alternative lending data and standard credit history evaluation, such as credit bureau ratings. We examine how transfer learning may help with credit scoring accuracy in this research. We investigate a domain with no or limited prior lending results, such as providing credit to unbanked or underbanked populations, or micro and small firms, where historical data is scarce. Lenders currently depend mostly on expert rules for credit scoring. Lenders demand a hefty fee or refuse to grant credit because of the considerable unpredictability of such scoring methods. Transfer learning from adjacent areas might help fill in the gaps in information and increase financial inclusion. Transferring CD loan knowledge to riskier small business loans, or utility bill payments to loan repayments, e.g., might result in a more accurate scoring model. The aim of this research is to address the following issue: can we use this “alternative lending” data to enhance credit behavior prediction, and hence credit access, for those with low traditional credit histories?

We explore how transfer learning could be used in the early stages of a credit risk model deployment when there is a lack of historical labeled data. The stability and accuracy of model performance in the credit risk domain are business goals in order to anticipate the chance of default. We provide a method for combining the results of transferred models from related credit risk domains with new models based on newly acquired labeled data from new domains. We get a greater level of precision while also maintaining the overall model's stability using this method. Experiments on real-world commercial data (which we are unable to discuss in this article) indicated that utilizing a gradually transitioned strategy, combining the transferred models and the new models can achieve these aims. We reproduce in this article versions of our experiments using publicly accessible Lending Club data to allow us to publish the results while still complying with the privacy standards of our client's data.

We focus on two scenarios in which a big dataset of current loan products is used to improve the credit risk model for new loan products with a considerably smaller dataset. In the first scenario, Lending Club data is used to resemble a lender that already possesses (CD) data and is ready to start lending to small companies. The second scenario likewise makes use of Lending Club data to resemble a lender that already has a credit card loan product and now wants to expand into vehicle loans.

For pre-processing, we pick 16 variables as inputs, and the output to be predicted is whether or not a loan will be approved or not. We transform the loan status to a binary result to simplify the model. Here, 1 indicates a defaulted loan, a charged-off loan, or a late loan payment; 0 indicates a paid-off debt. Current loans that are not yet due are not included in this calculation. The details of the pre-processing are detailed in Data Availability Statement.

Furthermore, to adopt a transfer learning approach in the real world, the following two questions need to be addressed. The first issue is to explain transferred models. Many jurisdictions require credit decisions to be explained for anti-discrimination and human rights purposes. For example, the European Union's General Data Protection Regulation (GDPR) requires “meaningful information about the logic involved” in automated decisions, providing an explanation that enables a data subject to exercise their rights under the GDPR and human rights law (Selbst and Powles, 2017). SHAP (Lundberg and Lee, 2017), based on Shapley values, is one of the most popular methods for explaining machine learned models. In this article, we apply SHAP to analyze the contribution of features and the impact of the transfer.

The second question is how to handle the difference between features from source and target domains. For instance, a source domain could be for a small short-term alternative loan, but the target domain may be for large and long-term installment loans. Key features, such as loan amount, loan terms, and interest cover. can differ between these domains. We could use the progressive shifting contribution network proposed in Suryanto et al. (2019) which combines source and target domain feature learning to improve model accuracy, but a key question that remains is: Can we adapt the features *before* transfer learning to get more accurate models?

We develop an approach to this question in this article based on three perspectives. First, we use a Kolmogorov Smirnov (KS) test to quantify the difference between source and target domains and use domain adaptation to treat only features that differ substantially between the domains before training. Second, after we find candidate features to be adapted, based on their KS differences, we include other features that are highly correlated with the candidate features and test the accuracy of models adapting these feature combinations. Finally, we exclude from adaptation those features related to a borrower's credit history where the adaptation would incorrectly impact the classification.

The remainder of the article is structured as follows. We cover related research in Section 2 and key aspects of the problem in Section 3. We describe our transfer learning approach in detail in Section 4, and in Section 5, we address two critical issues: domain adaptation and explainability. Discussion and conclusion are contained in the final two sections (6 and 7).

## 2. Related Studies

Transfer learning pre-dates deep learning (DL). Given the difficulty of defining features in image processing, many approaches were pioneered in that area. For example, the transfer of parameters from a trained SVM model was proposed by Yang et al. (2007). This can also be applied to unsupervised learning — a domain adaptation technique known as transfer component analysis was given by Pan et al. (2011). In their survey article, Pan et al. (2010) suggested four categories for transfer learning: transfer of instances, transfer of feature representations, transfer of parameter values, and transfer of relational knowledge.

Transfer learning requires training on a source domain and (re)training on a target domain, on which the class labels are to be predicted (for classification tasks). In this article, we are mainly concerned with the transfer of feature representations. The work on transductive transfer learning (Pan et al., 2011) has some similarities with our approach, but in that work, both the source and target classification tasks must be the same. A further difference in our approach is that we aim to progressively optimize for the right hyperparameter setting to balance the relative weight of the source to target the transfer of feature representations.

A central issue in transfer learning is the relation of the source to the target domain. This relationship can be affected by the relative heterogeneity of the data in the domain, and issues such as symmetry in the transfer of features, which can also impact the transfer of parameter values and relational knowledge, and the selection of the source domain, as discussed in Weiss et al. (2016). It is also important to be aware of and mitigate the potential for transfer learning to *decrease* performance on the target domain (which is known as “negative transfer”). Several approaches have been proposed to address this (Gao et al., 2008; Chattopadhyay et al., 2011; Sun et al., 2013; Xiao et al., 2014). However, in this study, we instead focussed on optimizing the architecture of target network models to enable the successful transfer of features. Addressing the risk of negative transfer in our approach is left for future study.

While the terms “transfer learning” and “domain adaptation” have been used interchangeably, we use transfer learning when the focus is the modeling configuration, and domain adaptation when the focus is on transforming the data. There are only a few published studies on domain adaptation for credit risk, e.g., Huang and Chen (2018) proposed domain adaptation for transforming the data distribution. In other domains, approaches such as Balanced Distribution Adaptation (Wang et al., 2017) and adapting without target labels have been used (Huang and Chen, 2018; Zhang et al., 2018; Kouw and Loog, 2019).

In this article, we use a method of Progressive Network configuration for transfer learning, similar to Rusu et al. (2016). The contribution of this article is a strategy to apply domain adaptation to the source data when target data with labels is limited and to apply both domain adaptation and transfer learning to credit risk. Neyshabur et al. (2020) investigated what is being transferred, which are general features in the lower layers and more specific features in the higher layers. Our Progressive Network configuration facilitates the search to find from which layers we retrain the network. In credit risk, where the data labels are scarce, the approach such as transferring learned knowledge from self-supervised tasks to downstream tasks could improve the performance of the network (Han et al., 2021).

## 3. Credit Risk

Across their loan portfolios, lenders strive to maximize the risk-return ratio. The cornerstone of this optimization is the accurate and consistent measurement of credit risk. Expected Loss (*EL*) is a standard metric used by lenders to assess credit risk. *EL* is mostly governed by the Probability of Default (*PD*) in an unsecured loan scenario. *PD* is calculated using credit scoring models. The characteristics of the loan applicant and their application are usually inputs to a credit rating algorithm. To demonstrate our techniques, we use attributes from lendingclub.com data. The most often used metrics in credit risk assessment include the Gini Index, KS statistics, Lift, Mahalanobis distance, and information statistics (Řezáč and Řezáč, 2011). The Gini Index (abbreviated as "Gini") is used in this study.

A score ranging from 0 to 1 is generated by the scoring model. It is a likelihood of default that has been calculated. Typically, a portion of the data is pre-allocated for score calibration. With the resulting *PD* and loan application data as inputs, lenders utilize a set of decision processes and rules to make the best conclusion possible. An eligibility test is usually the first step in the decision-making process. The *PD* is calculated for all potential candidates and then used to divide them into decision groups. The interest rate, e.g., may differ depending on the decision group, as could the loan amount as a proportion of net income.

The focus of this study is on credit rating for unsecured loans. The Area Under Receiver Operating Curve (*AUC*) or *GiniROC*, which is 2*AUC*−1 (Flach, 2003), is used to evaluate the performance of our credit scoring model. The Gini Index, which is employed as the splitting criteria in CART (Breiman, 1996), is based on the same principle as GiniROC. Gini and GiniROC, on the other hand, serve distinct purposes. The measure GiniROC is used to assess model quality based on *PD* without the requirement to transform *PD* into binary classifications because the threshold for doing such classifications is established in the credit decisioning.

### 3.1. Credit Scoring

Credit scoring generates a *PD* value that is used to estimate whether a loan will be repaid or defaulted. There are other possible consequences in real-world settings, such as late or incomplete payment. We need a measure to assess the model's quality without setting a threshold to transform the *PD* into a classification in credit scoring. We can use a statistic like *Fscore* after we have these classifications. This judgment is delayed in credit risk to the credit-decisioning process when expert rules/guidelines are used to determine whether or not the loan is accepted.

### 3.2. Credit Decisioning

Credit decisioning uses *PD* to accept or deny a loan application. A mapping table is used to map ranges of *PD* to decisions when converting from *PD* to decisions. It may also change the loan amount, interest rate, and period, in addition to approving or declining the loan. Because the data is typically scarce and/or the search space is too big for supervised learning models to be built, this model is typically based on expert rules.

## 4. Transfer Learning

In this section, we first outline our transfer learning framework and then summarize the experimental results (derived from those of Suryanto et al., 2019). The key idea of this part of our study is the notion of Progressive Shift Contribution (*PSC*) where a combination of network architecture operators and training methods enable a number of variants of transfer learning to be implemented and evaluated. Essentially, the idea behind *PSC* is to generate different transfer learning models in which the target domain contribution gradually grows while the source domain contribution drops. We first describe a base model network architecture, explained in Section 4.2, then how to create a variety of network topologies from this in Section 4.3.1. We empirically evaluate the effectiveness of this approach to transfer learning algorithms by testing each variant model on different source and target domain datasets, with the results shown in Section 4.4.

### 4.1. Model Development

A total of six different network architectures were developed, which we refer to in the following as Models 1–6.

Only source data is used to train Model No. 1. We progressively shifted the domain contribution from source to target domain in Model Nos. 2–5. Only target domain data is used to train Model No. 6, the last model. The ratio of trained layers using the target domain to trained layers using the source domain indicates the contribution variations between the source and target domains. This approach can be generalized to a network configuration of any size. The algorithm's specifics will be explored in further depth in Section 4.3.1. All experiments' source code and data are accessible in Data Availability Statement.

To find the optimum network configuration, we transferred the *PSC* from the source domain to the target domain and evaluated Gini performance using test data from the target domain. The performance of the model is primarily determined by the following factors:

a) methodologies used for modeling (e.g., generalized linear model, gradient boosting machine, deep learning), hyper parameters^2^, b) data signal strength, and c) feature engineering. We can express the link between *Gini*, which we denote by 𝔾, and the above factors as follows:


(1) $$\begin{array}{r} 𝔾=𝔤(T\left({𝔐}_{𝔢},{𝔰}_{𝔢}\right) \end{array}$$

where 𝔰_𝔢_ represents the source domain's test data, 𝔐_𝔢_ represents the model trained on the source domain's training data, T() represents th activity of testing a model on the test data that produces the test results, and 𝔤() represents a function to compute the Gini coefficient of the results. The variable 𝔐_𝔢_ is defined as follows:


(2) $$\begin{array}{r} {𝔐}_{𝔢}=T_{N}\left({𝔐}_{0},{𝔓}_{𝔢},{𝔱}_{𝔢},{𝔉}_{𝔢}\right) \end{array}$$

where 𝔐_*0*_ is a configuration of a deep neural network with initial random weights, 𝔓_𝔢_ is a set of hyper parameters for training 𝔐_𝔢_, 𝔱_𝔢_ is the source domain's training data, 𝔉_𝔢_ is a collection of features obtained from 𝔱_𝔢_. $T_{N}$() is an activity to train a model with these four factors. The outcome of $T_{N}$() is a model that has been trained using the above four factors.

We now describe how *PSC* is performed. To begin, we define a function S() that partitions 𝔐_𝔢_ into two segments: 𝔐_𝔛_𝔢__ and 𝔐_𝔉_𝔢__. 𝔐_𝔛_𝔢__ denotes the segment in which the layers were trained using 𝔱_𝔢_ and the layers are not re-trainable. 𝔐_𝔉_𝔢__ is the segment in which the layers were also trained using 𝔱_𝔢_, however, these layers can be re-trained using the target domain's training data 𝔱_𝔫_.


(3) $$\begin{array}{r} \left({𝔐}_{{𝔛}_{𝔢}},{𝔐}_{{𝔉}_{𝔢}}\right)=S\left({𝔐}_{𝔢}\right) \end{array}$$

S()'s inverse function is C(), which combines 𝔐_𝔛_𝔢__ and 𝔐_𝔉_𝔢__ to form 𝔐_𝔢_.


(4) $$\begin{array}{r} {𝔐}_{𝔢}=C\left({𝔐}_{{𝔛}_{𝔢}},{𝔐}_{{𝔉}_{𝔢}}\right) \end{array}$$

For the target domain 𝔐_𝔱*𝔯𝔞𝔫𝔰𝔣𝔢𝔯*_, we developed a model, that incorporates data from both the source and target domains, by transferring the structure and weights of the 𝔐_𝔛_𝔢__ layers and retraining the 𝔐_𝔉_𝔢__ layers.


(5) $$\begin{array}{r} {𝔐}_{{𝔉}_{𝔫}}=T_{N}\left({𝔐}_{{𝔉}_{𝔢}},{𝔓}_{𝔫},{𝔱}_{𝔫},{𝔉}_{𝔫}\right) \end{array}$$

In the end, we put together the target model 𝔐_𝔉_𝔫__ and the model 𝔐_𝔛_𝔢__. The result is the transferred model, which is called 𝔐_𝔱*𝔯𝔞𝔫𝔰𝔣𝔢𝔯*_.


(6) $$\begin{array}{r} {𝔐}_{𝔱𝔯𝔞𝔫𝔰𝔣𝔢𝔯}=C\left({𝔐}_{{𝔛}_{𝔢}},{𝔐}_{{𝔉}_{𝔫}}\right) \end{array}$$

The overarching objective is to maximize 𝔾_𝕋_ by tracking 𝔾_𝕋_ as the *PSC* moves from source to target domain data. We evaluate the performance of all six network topologies in Section 4.1 to get the highest 𝔾_𝕋_. In the following equation, 𝔰_𝔫_ represents the target domain's test data.


(7) $$\begin{array}{r} {𝔾}_{𝕋}=𝔤\left(T\left({𝔐}_{𝔱𝔯𝔞𝔫𝔰𝔣𝔢𝔯},{𝔰}_{𝔫}\right)\right) \end{array}$$

### 4.2. The Base Model

**Figure 1:**
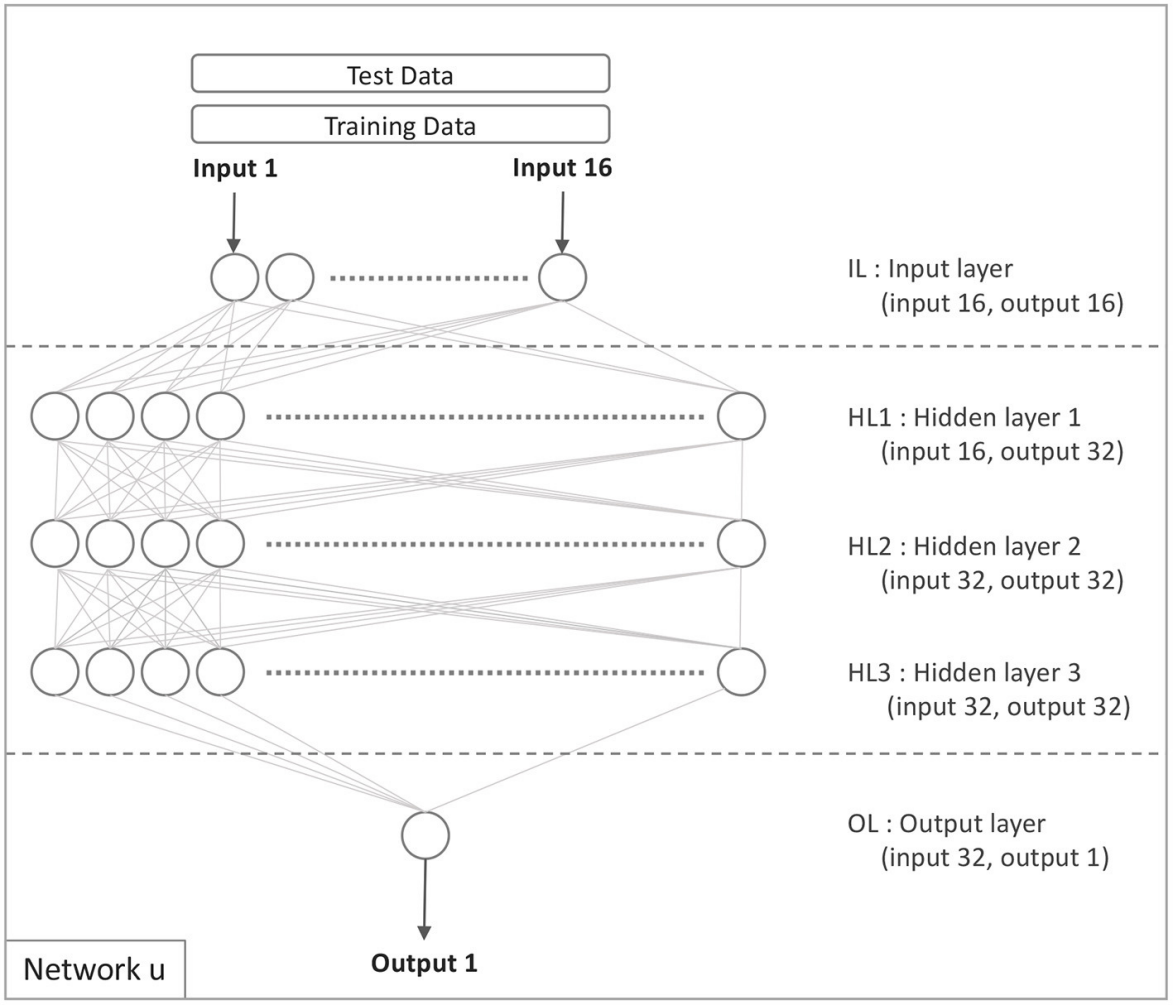
Network u: the base model.

Figure 1 illustrates the initial basic model. It contains 16 input nodes on the input layer, three hidden layers with 32 nodes on each, and one output node on the output layer. The configuration of the network is chosen using a hyper parameter search to get a configuration that is close to optimal. By and large, the base models were constructed in accordance with network architectures.

### 4.3. The Comparison Model (Model u)

Figure 1 illustrates the network configuration u on which the comparison model is based. Only the target domain data is used to train this model, no data from the source domain is utilized. The model developed using target domain data is defined similarly to Equation 2:


(8) $$\begin{array}{r} 𝔐{\left(𝔲\right)}_{𝔫}=T_{N}\left(𝔐{\left(𝔲\right)}_{0},{𝔓}_{𝔫},{𝔱}_{𝔫},{𝔉}_{𝔫}\right) \end{array}$$

where 𝔐(𝔲)_𝔫_ is a model based on network configuration u that was built using data from the target domain. The starting model 𝔐(𝔲)_*0*_ is based on network configuration u with all weights randomly initialized, and the parameters, training data, and features required to build the model 𝔐(𝔲)_𝔫_ are 𝔓_𝔫_, 𝔱_𝔫_, and 𝔉_𝔫_.


(9) $$\begin{array}{r} 𝔾=𝔤\left(T\left(𝔐{\left(𝔲\right)}_{𝔫},{𝔰}_{𝔫}\right)\right) \end{array}$$

In the above equation, *s*_*n*_ denotes the target domain's test data.

#### 4.3.1. PSC Models

Six models were introduced in Section 4.1, where the *PSC* changes between the source and target domain data. We added an extra parameter to the split function provided in Equation 3 to determine the fraction of *PSC*. This parameter's value is one of the following: *v*, *N*_1_, *N*_1_*N*_2_, *N*_1_*N*_2_*N*_3_, or *N*_1_*N*_2_*N*_3_*N*_4_. Based on the varied *PSC* from the source to the target domain, each value results in a distinct network configuration. For each of these five values, we created five *PSC* models. In addition, we include the baseline Comparison Model mentioned in Section 4.3. In the next sections, we go over Models 2 through 6.

#### 4.3.2. Model *v*

**Figure 2:**
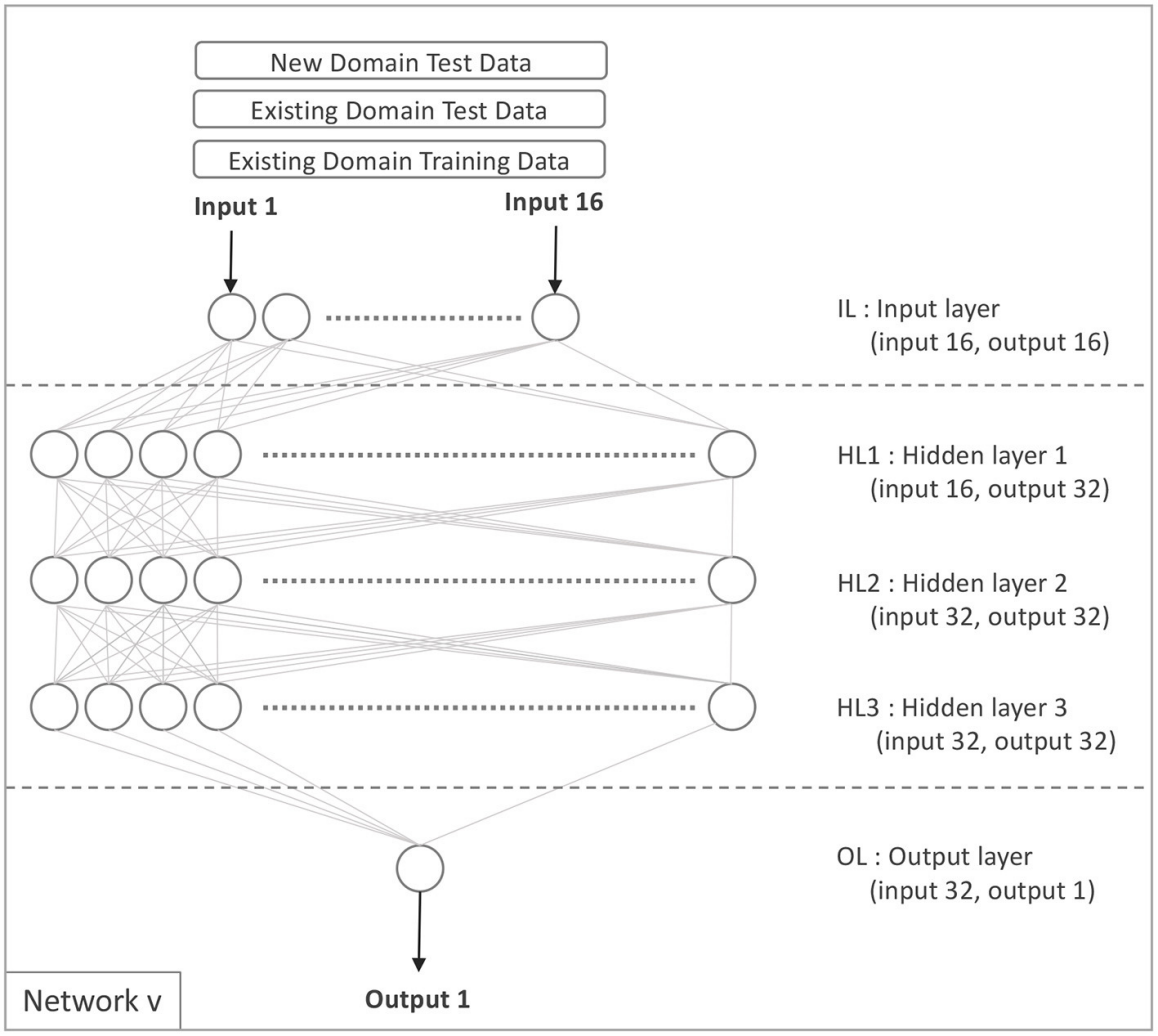
Network v.

Model *v* is exclusively constructed from the source domain data only. To generate Model *v*, we first trained model 𝔐(𝔳)_𝔢_ using Equation 10 and the network configuration shown in Figure 2.


(10) $$\begin{array}{r} 𝔐{\left(𝔳\right)}_{𝔢}=T_{N}\left(𝔐{\left(𝔳\right)}_{0},{𝔓}_{𝔢},{𝔱}_{𝔢},{𝔉}_{𝔢}\right) \end{array}$$

On target domain data, the model was evaluated, and a Gini coefficient was computed.


(11) $$\begin{array}{r} 𝔾=𝔤\left(T\left(𝔐{\left(𝔳\right)}_{𝔢},{𝔰}_{𝔫}\right)\right) \end{array}$$

#### 4.3.3. Model *N*_1_*N*_2_*N*_3_*N*_4_

Four parallel networks were used to develop this model, each with three hidden layers connected to the input and output layers. To begin, we replicated the hidden layers of network v into networks *N*_1_, *N*_2_, *N*_3_, and *N*_4_. We explain the transformation conceptually using Equation 12.


(12) $$\begin{array}{r} 𝔐{\left(N_{1}N_{2}N_{3}N_{4}\right)}_{𝔢}=TRANSFORM\left(𝔐{\left(𝔳\right)}_{𝔢}\right) \end{array}$$

**Figure 3:**
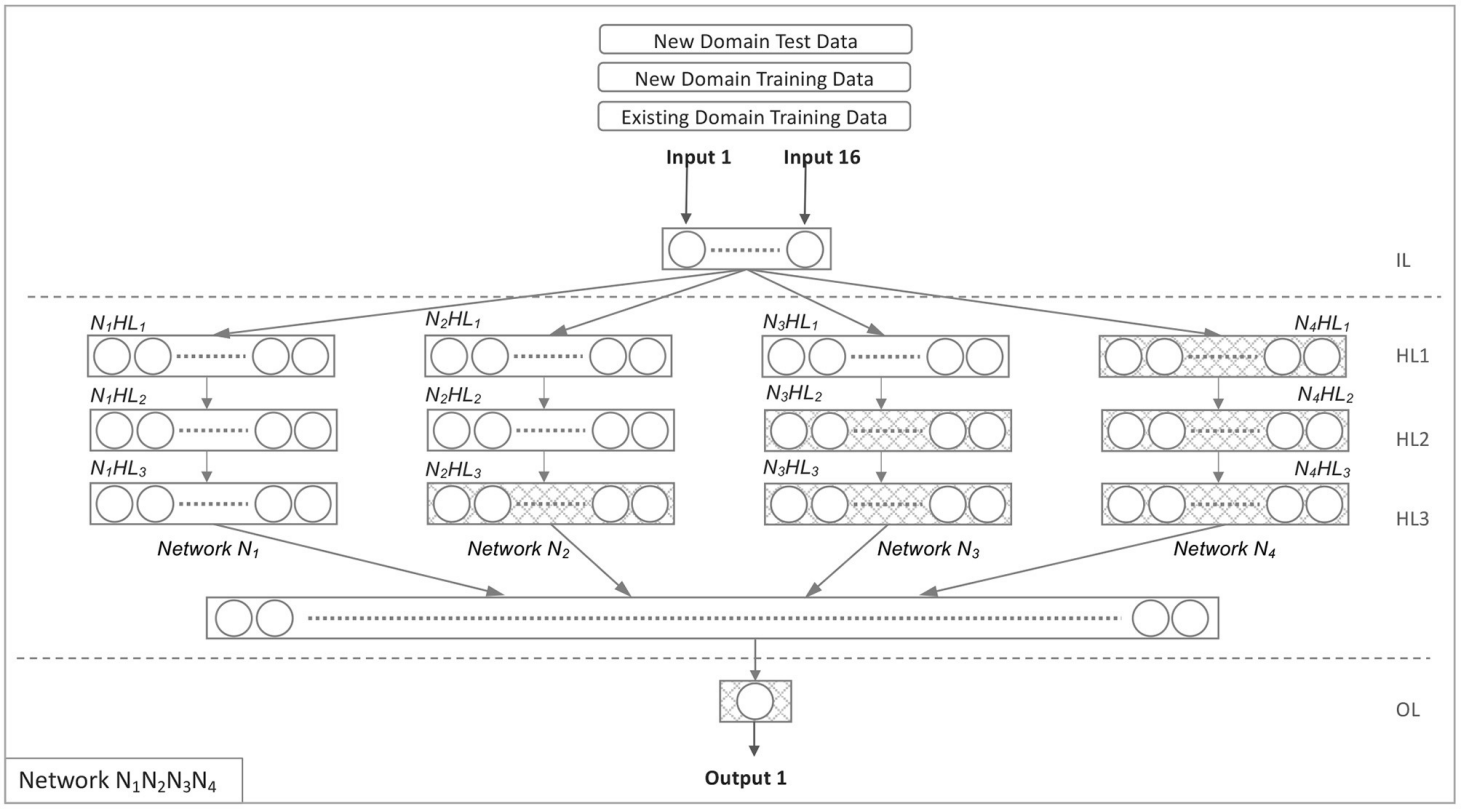
Network *N*_1_*N*_2_*N*_3_*N*_4_.

The networks *N*_1_, *N*_2_, *N*_3_, and *N*_4_ were configured in the manner depicted in Figure 3.

After the structure and weights were set (as shown in Figure 3), we then set the following as trainable, using the target domain data: The 3rd hidden layer of Network *N*_2_, the 2nd and 3rd hidden layers of Network *N*_3_, and all hidden layers of Network *N*_4_. The next three steps are indicated in numbers 1, 2, 3 within ellipses in Figure 3:

Following the establishment of the structure and weights (as seen in Figure 3), we designated the following hidden layers as trainable using the target domain data: *N*_2_*HL*_3_, *N*_3_*HL*_2_, *N*_3_*HL*_3_, *N*_4_*HL*_1_, *N*_4_*HL*_2_, and *N*_4_*HL*_3_. The next three steps are as below:

1. The source domain's training data (𝔱_𝔢_) is used to derive weights for all four networks (*N*_1_, *N*_2_, *N*_3_, and *N*_4_). Depending on the configuration, some hidden layers in networks *N*_1_, *N*_2_, *N*_3_, and *N*_4_ and the output layer are configured to be re-trainable, using the target domain's training data (𝔱_𝔫_).
2. Train re-trainable layers using the target domain's training data (𝔱_𝔫_).
3. Evaluate the whole parallel network (*N*_1_, *N*_2_, *N*_3_, and *N*_4_) using the target domain's test data (𝔰_𝔫_), and then compute the Gini coefficient from the result.

The following three equations can describe the evolution of Model *N*_1_*N*_2_*N*_3_*N*_4_:


(13) $$\begin{array}{r} \left({𝔐}_{𝔛}{\left(N_{1}N_{2}N_{3}N_{4}\right)}_{e},{𝔐}_{𝔉}{\left(N_{1}N_{2}N_{3}N_{4}\right)}_{e}\right)=S\left(𝔐{\left(N_{1}N_{2}N_{3}N_{4}\right)}_{e}\right) \end{array}$$

(14) $$\begin{array}{r} {𝔐}_{𝔉}{\left(N_{1}N_{2}N_{3}N_{4}\right)}_{n}=T_{N}\left({𝔐}_{𝔉}{\left(N_{1}N_{2}N_{3}N_{4}\right)}_{e},{𝔓}_{𝔫},{𝔱}_{𝔫},{𝔉}_{𝔫}\right) \end{array}$$

(15) $$\begin{array}{r} 𝔐{\left(N_{1}N_{2}N_{3}N_{4}\right)}_{transfer}=C\left({𝔐}_{𝔛}{\left(N_{1}N_{2}N_{3}N_{4}\right)}_{e},{𝔐}_{𝔉}{\left(N_{1}N_{2}N_{3}N_{4}\right)}_{n}\right) \end{array}$$

Model *N*_*1*_*N*_*2*_*N*_*3*_*N*_*4*_ was trained using the source domain data and six hidden layers and the output layer was retrained using the target domain data.

#### 4.3.4. Model *N*_1_

**Figure 4:**
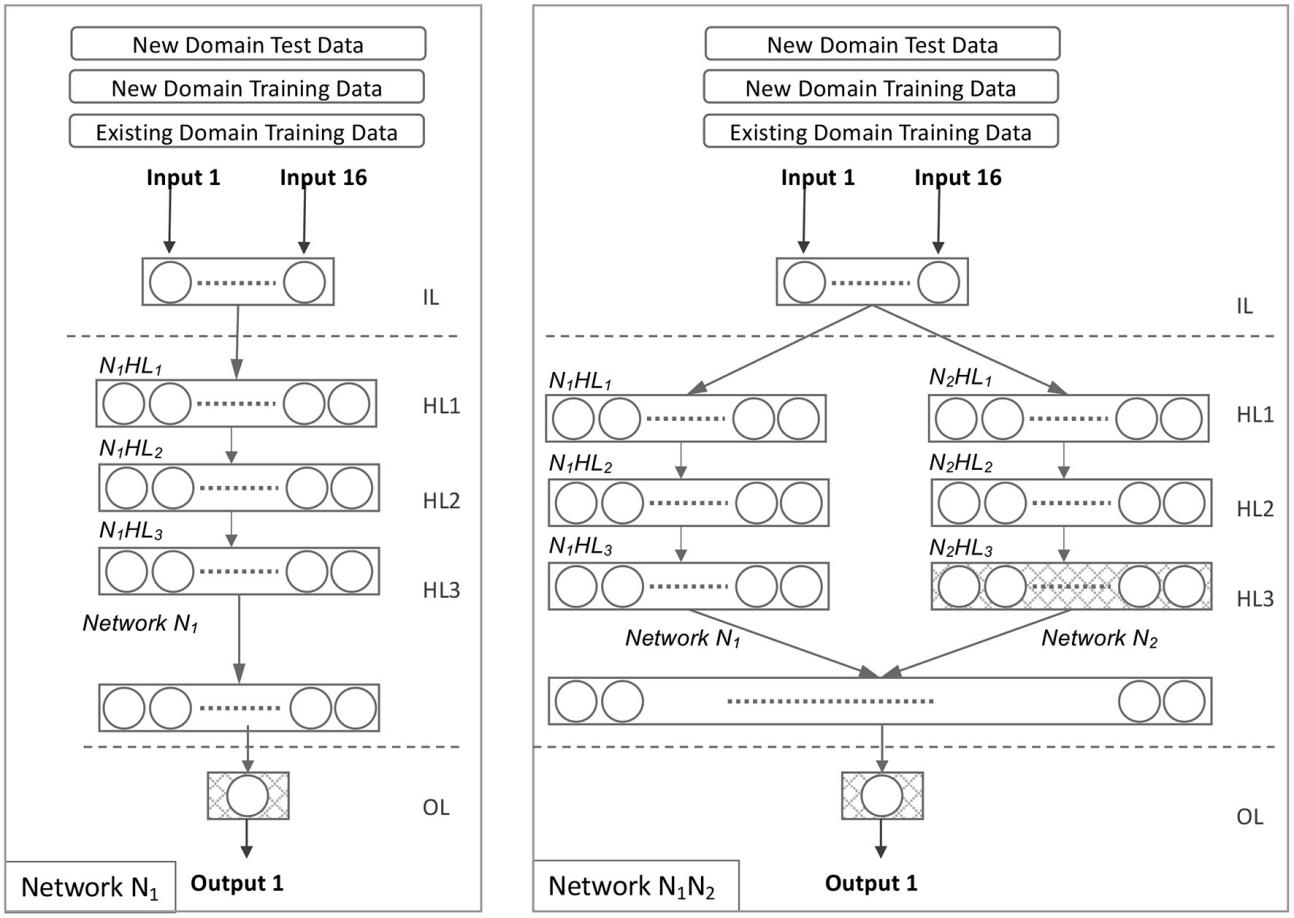
Network *N*_1_ and Network *N*_1_*N*_2_.

After deleting Networks *N*_2_, *N*_3_, and *N*_4_ from the model *N*_1_*N*_2_*N*_3_*N*_4_, we derive Model *N*_1_. Figure 4 illustrates this network configuration. All hidden layers were trained on source domain data and only the output layer was retrained on target domain data in Model *N*_1_. Equations 16, 17, and 18 illustrate the evolution of Model *N*_1_.


(16) $$\begin{array}{r} \left({𝔐}_{𝔛}{\left(N_{1}\right)}_{e},{𝔐}_{𝔉}{\left(N_{1}\right)}_{e}\right)=S\left(𝔐{\left(N_{1}\right)}_{e}\right) \end{array}$$

(17) $$\begin{array}{r} {𝔐}_{𝔉}{\left(N_{1}\right)}_{n}=T_{N}\left({𝔐}_{𝔉}{\left(N_{1}\right)}_{e},{𝔓}_{𝔫},{𝔱}_{𝔫},{𝔉}_{𝔫}\right) \end{array}$$

(18) $$\begin{array}{r} 𝔐{\left(N_{1}\right)}_{transfer}=C\left({𝔐}_{𝔛}{\left(N_{1}\right)}_{e},{𝔐}_{𝔉}{\left(N_{1}\right)}_{n}\right) \end{array}$$

#### 4.3.5. Model *N*_1_*N*_2_

Model *N*_1_*N*_2_ is derived from Model *N*_1_*N*_2_*N*_3_*N*_4_ by excluding Networks *N*_3_ and *N*_4_. Figure 4 illustrates this network configuration. All hidden layers were trained on the source domain data in Model *N*_1_*N*_2_. The target domain data was used to retrain one hidden layer (*N*_2_*HL*_3_) and the output layer. The evolution of the model *N*_1_*N*_2_ is illustrated in Equations 19, 20, and 21.


(19) $$\begin{array}{r} \left({𝔐}_{𝔛}{\left(N_{1}N_{2}\right)}_{e},{𝔐}_{𝔉}{\left(N_{1}N_{2}\right)}_{e}\right)=S\left(𝔐{\left(N_{1}N_{2}\right)}_{e}\right) \end{array}$$

(20) $$\begin{array}{r} {𝔐}_{𝔉}{\left(N_{1}N_{2}\right)}_{n}=T_{N}\left({𝔐}_{𝔉}{\left(N_{1}N_{2}\right)}_{e},{𝔓}_{𝔫},{𝔱}_{𝔫},{𝔉}_{𝔫}\right) \end{array}$$

(21) $$\begin{array}{r} 𝔐{\left(N_{1}N_{2}\right)}_{transfer}=C\left({𝔐}_{𝔛}{\left(N_{1}N_{2}\right)}_{e},{𝔐}_{𝔉}{\left(N_{1}N_{2}\right)}_{n}\right) \end{array}$$

#### 4.3.6. Model *N*_1_*N*_2_*N*_3_

**Figure 5:**
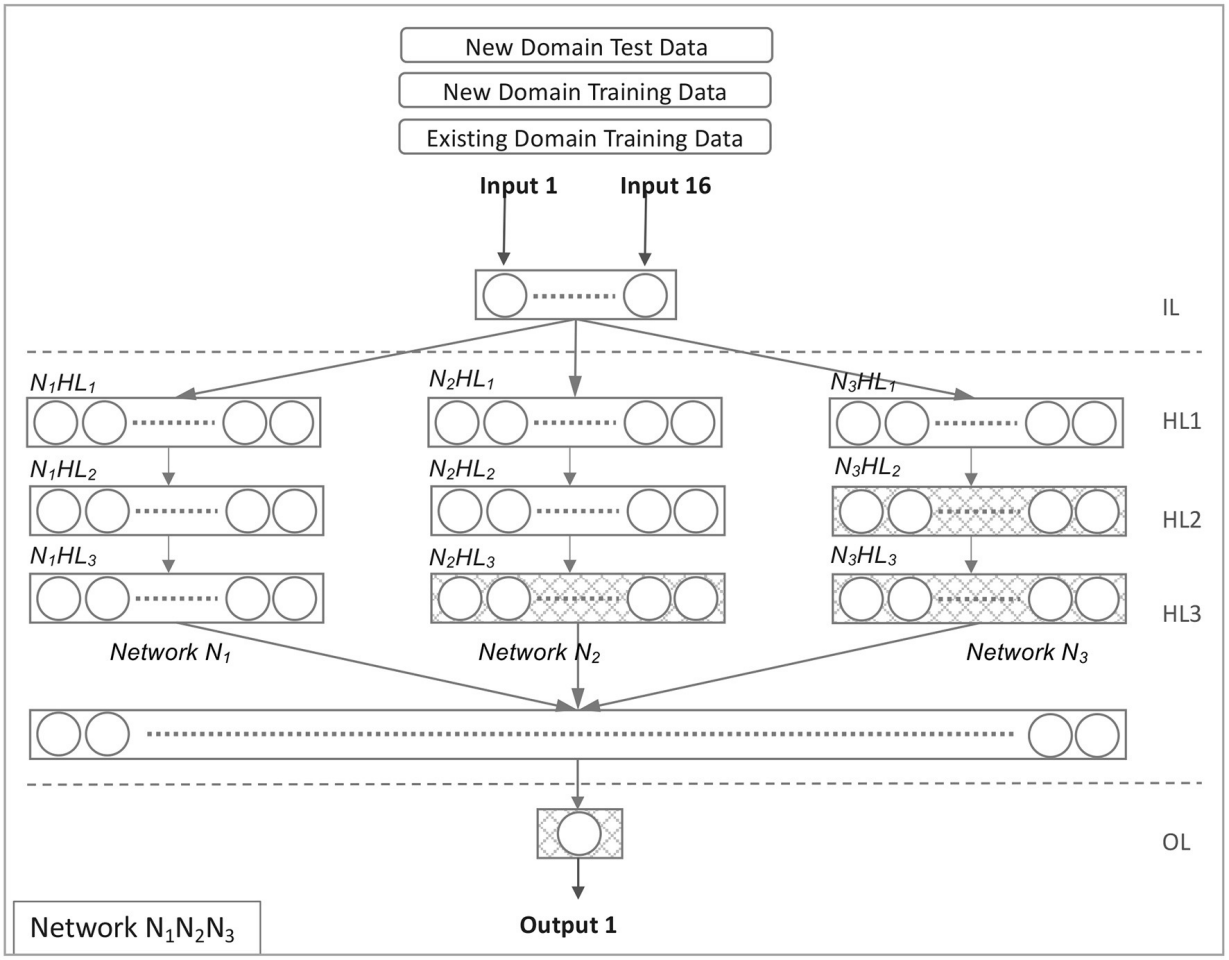
Network *N*_1_*N*_2_*N*_3_.

Model *N*_1_*N*_2_*N*_3_ is derived from Model *N*_1_*N*_2_*N*_3_*N*_4_, excluding Network *N*_4_. Figure 5 illustrates this network configuration. All hidden layers were trained in Model *N*_1_*N*_2_*N*_3_ using source domain data. The target domain data was used to retrain three hidden layers (*N*_2_*HL*_3_, *N*_3_*HL*_2_, and *N*_3_*HL*_3_) and the output layer. Equations 22, 23, and 24 illustrate the evolution of the model *N*_1_*N*_2_*N*_3_.


(22) $$\begin{array}{r} \left({𝔐}_{𝔛}{\left(N_{1}N_{2}N_{3}\right)}_{e},{𝔐}_{𝔉}{\left(N_{1}N_{2}N_{3}\right)}_{e}\right)=S\left(𝔐{\left(N_{1}N_{2}N_{3}\right)}_{e}\right) \end{array}$$

(23) $$\begin{array}{r} {𝔐}_{𝔉}{\left(N_{1}N_{2}N_{3}\right)}_{n}=T_{N}\left({𝔐}_{𝔉}{\left(N_{1}N_{2}N_{3}\right)}_{e},{𝔓}_{𝔫},{𝔱}_{𝔫},{𝔉}_{𝔫}\right) \end{array}$$

(24) $$\begin{array}{r} 𝔐{\left(N_{1}N_{2}N_{3}\right)}_{transfer}=C\left({𝔐}_{𝔛}{\left(N_{1}N_{2}N_{3}\right)}_{e},{𝔐}_{𝔉}{\left(N_{1}N_{2}N_{3}\right)}_{n}\right) \end{array}$$

### 4.4. Experiments

We utilized data from lendingclub.com in our studies, which is similar to our original client's (confidential) data, and spans the years 2007–2018 (refer to Data Availability Statement). We began by developing base models and training them without using transfer learning. A grid search was then used to identify a close-to-optimal set of hyper-parameters for each neural network architecture. To evaluate our transfer learning approach, we selected data on *CD* as the source domain and Small Business (*SB*) as the target domain. Our objective is to evaluate the implementation of transfer learning from *CD* to *SB*, to exemplify a real-world situation where data in the target domain (here *SB*) is scarce, but data in the source domain (here *CD*) is more readily available.

To validate the performance of *DL* on the *CD* and *SB* datasets, we also compared it to that of Gradient Boosting Machines (*GBM*). The Gini coefficients on the *CD* data were equal (0.43, with a s.d. of 0.01). On the *SB* data, they were almost identical, at 0.30 with 0.05 s.d. for *GBM* compared to 0.31 with 0.02 s.d. for *DL*, indicating that there is no statistically significant difference in performance between the two methods. Consequently, in the following sections, our experiments concentrate exclusively on *DL*.

#### 4.4.1. Datasets

From the data acquired from lendingclub.com, ten datasets were generated. For the first source domain dataset, *CD*4, we randomly selected 1,00,000 records from 9,40,948 records on loans for the purpose of paying off credit cards and consolidating debt. The rate of bad debt in this sample was 21%. The first target domain dataset, *SB*4, had 13,794 records pertaining to loans made for the purpose of investing in small businesses. This is a more risky loan type, where the default rate is 30%. These two datasets were not subjected to any outlier screening.

We then constructed the source domain datasets *CD*1, *CD*2, and *CD*3 as subsets of the dataset *CD*4, with different time range filters applied. Target domain datasets *SB*1, *SB*2, and *SB*3 were also constructed as subsets of *SB*4 similarly to those for the source domain. These restrictions allow investigation of any effects due to changes in the time range of the datasets used.

Additionally, we identified a further pair of source and target domain datasets, as follows. The source domain *CCD* is a subset of the *CD*4 dataset that was filtered to extract Credit Card Loans. Similarly, the Car Loan data target domain dataset was a set of car loans extracted from Lending Club datasets. Both these datasets both spanned the time range 2007–2018, as in *CD*4 and *SB*4 (for further details refer to the link provided in Data Availability Statement).

The data in Table 1 was used in all of the experiments. They were performed using a 10-fold cross-validation procedure that was done five times. The basic model for the transfer learning was created using the datasets *CD*1, *CD*2, *CD*3, *CD*4, and *CCD*, and the network configuration *u*, as illustrated in Figure 1 and specified in Equation 2. The intensity of the signal associated with the result being predicted from the data was one element that affected model performance.

**Table 1:** The datasets used in the transfer learning studies are listed below.

**ID**	**Dataset**	**Period**	**Size**	**Type**	**Gini (s.d.)**
CD1	*CreditCard*/*DebtConsolidation*	2007–2011	23,813	Source	0.364(0.023)
SB1	*SmallBusinessLoan*	2007–2011	1,831	Target	0.272(0.067)
CD2	*CreditCard*/*DebtConsolidation*	2007–2014	100,000	Source	0.417(0.016)
SB2	*SmallBusinessLoan*	2007–2014	6,686	Target	0.274(0.040)
CD3	*CreditCard*/*DebtConsolidation*	2007–2016	100,000	Source	0.447(0.013)
SB3	*SmallBusinessLoan*	2007–2016	12,114	Target	0.331(0.032)
CD4	*CreditCard*/*DebtConsolidation*	2007–2018	100,000	Source	0.448(0.012)
SB4	*SmallBusinessLoan*	2007–2018	13,794	Target	0.351(0.024)
CCD	*CreditCard*	2007–2018	100,000	Source	0.463(0.014)
CAR	*CarLoan*	2007–2018	12,734	Target	0.436(0.036)

*The “Type” column shows whether the dataset is used as the source or target for the transfer learning process; the results given are cross-validation runs' means and standard deviations (s.d.)*.

As seen in Table 1, there is an effect of both dataset size (on the target domain, the larger the dataset, and the higher the Gini) and the date range (on the source domain, the more time covered by the dataset, the higher the Gini) on the baseline performance.

As in Equation 9, the Gini values for *SB*1, *SB*2, *SB*3, *SB*4, and *CAR* (as shown in Tables 1, 2) are computed from the test result of model *M*(*u*)_*n*_ by applying the function *g*() to the test results (for source domain datasets we use model *v*).

**Table 2:** Experimental Results for six models with progressively shifted contribution, built on the source and target datasets described in Table 1 (all of the source:Credit Card/Debt Consolidation (CD), target:Small Business (SB) Loan datasets, plus the source:Credit Card, target:Car Loan datasets); results shown as *means (s.d.)*; models with the highest performance in each column are denoted by the symbol *.

	**Source/Target**				
**Model**	***CD*1/*SB*1**	***CD*2/*SB*2**	***CD*3/*SB*3**	***CD*4/*SB*4**	***CCD*/*CAR***
*M*(*v*)_*e*_	0.157(0.022)	0.236(0.051)	−0.191(0.260)	0.196(0.026)	0.262(0.355)
*M*(*N*1)_*transfer*_	*0.301(0.097)	*0.287(0.051)	0.334(0.029)	0.350(0.029)	*0.447(0.037)
*M*(_*N*_1_*N*_2_)*transfer*_	0.292(0.091)	0.272(0.054)	*0.337(0.030)	0.350(0.028)	0.434(0.035)
*M*(_*N*_1_*N*_2_*N*_3_)*transfer*_	0.230(0.087)	0.217(0.057)	0.300(0.032)	0.310(0.030)	0.376(0.040)
*M*(_*N*_1_*N*_2_*N*_3_*N*_4_)*transfer*_	0.174(0.010)	0.172(0.051)	0.254(0.029)	0.273(0.030)	0.310(0.050)
*M*(*u*)_*n*_	0.272(0.067)	0.274(0.040)	0.331(0.032)	*0.351(0.024)	0.436(0.036)
% improvement	10.7%	4.7%	1.8%	0.0%	2.5%

#### 4.4.2. Results

The experimental findings are reported in Table 2, in which we applied Progressive Shifted Contributions (*PSC*), moving from the source domain to the target domain data. All of these experiments were carried out using a 10-fold cross-validation procedure that was repeated five times. In other words, each experiment was performed 50 times, and the mean of the Gini scores and the standard deviation (denoted s.d. in tables) were recorded.

For the experiment over source/target *CD*1/*SB*1, we see that *M*(_*N*_1_)*transfer*_ had the greatest Gini of 0.301, an improvement of 10.7% over *M*(*u*)_*n*_. As the *CD*2/*SB*2 target data covers a wider time range and has more examples, *M*(_*N*_1_)*transfer*_ retained the highest Gini of 0.287, but the improvement was just 4.7 percent. As *CD*3/*SB*3 target domain time range increased further along with further increases in dataset size, the contribution moved toward the target domain; model *M*(_*N*_1_*N*_2_)*transfer*_ had the greatest Gini of 0.337, however, with just a minor improvement of 1.8% above *M*(*u*)_*n*_. Finally, the contribution was totally moved toward the target domain in *CD*4/*SB*4, resulting in the highest Gini coefficient of 0.351 for *M*(*u*)_*n*_.

From the experiments conducted on the datasets *CCD* and *CAR*, *M*(_*N*_1_)*transfer*_ produced the highest Gini score of 0.447, representing a slight improvement (2.5%) over the previous experiment, *M*(*u*)_*n*_. This may be due to a greater similarity between source and target domains for *CCD*/*CAR* than that for *CD*/*SB*, which could be investigated as part of further study. However, it also demonstrates that the progression from the source to target data finding the best balance improves the quality of transfer learning.

From the experimental results, it is evident that the effect of progression from source to target has more impact as data is selectedfrom a wider time range in both the source and target domains. The contribution was dependent on the number of re-trainable layers.

#### 4.4.3. Additional Experiments

We explored the possibility that the Gini improvement was attributable to the complexity of the network's structure. We ran experiments in accordance with Equations 25 and 26. On source domain data, the model with network configuration *N*_1_*N*_2_*N*_3_*N*_4_ was trained and retrained. This model's performance was 0.39 (0.01), which was lower than the base model Gini of 0.43 (0.01). This demonstrates that the added complexity of *M*(_*N*_1_*N*_2_*N*_3_*N*_4_)*transfer*_ has no effect on Gini performance. As a result, the improvement must be attributable to the diversity of the source data, which supplements the target data.


(25) $$\begin{array}{r} {𝔐}_{𝔉}{\left(N_{1}N_{2}N_{3}N_{4}\right)}_{e}=T_{N}\left({𝔐}_{𝔉}{\left(N_{1}N_{2}N_{3}N_{4}\right)}_{e},{𝔓}_{𝔢},{𝔱}_{𝔢},{𝔉}_{𝔢}\right) \end{array}$$

(26) $$\begin{array}{r} 𝔐{\left(N_{1}N_{2}N_{3}N_{4}\right)}_{retrain}=C\left({𝔐}_{𝔛}{\left(N_{1}N_{2}N_{3}N_{4}\right)}_{e},{𝔐}_{𝔉}{\left(N_{1}N_{2}N_{3}N_{4}\right)}_{e}\right) \end{array}$$

#### 4.4.4. Transfer Learning: Summary

We have presented an algorithm for gradually shifting the contribution from the source domain to the target domain. We can assess incremental complements of target domain data with source domain data using the *PSC* algorithm. While certain tasks were done manually, the overarching purpose was to create a framework that can automatically search for the optimum balance of source and target domain data, resulting in the highest Gini score for that combination. Models ranging from Model *v* (using just source domain data) to Model *u* (using only target domain data) were created. These empirical results have shown that the method of transfer learning developed in this article can be applied in the area of model-based credit risk assessment. Furthermore, the results demonstrate that our method is capable of optimizing over a structured series of network architectures to find the best balance between the contribution of source and target domains.

## 5. Domain Adaptation and Explainability

In this section, we address the two important questions of domain adaptation and explainability.

### 5.1. Explainability

We use SHapley Additive exPlanations (SHAP)^3^ to calculate the feature contributions for source models, target models, and transferred models. Source models are trained and tested on source domain data, using the same neural network configuration as the target models. The sole purpose of the source models is to investigate the feature contributions of the source domain to compare with transferred models.

**Figure 6:**
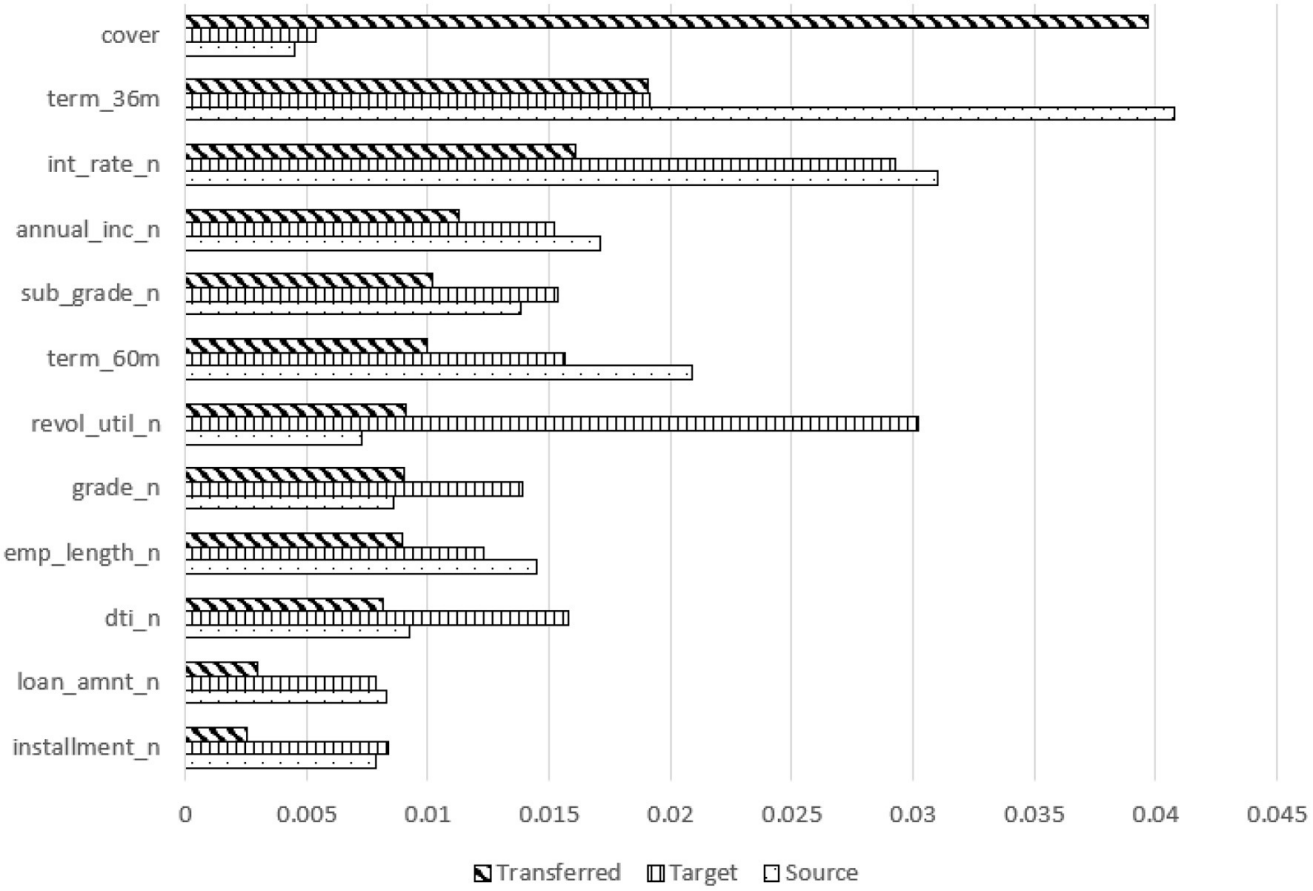
Feature contribution comparison for transferred, target, and source models using SHAP-transferring from CD to MD.

We start by constructing a neural network model using training data, then feed this model and the training data into SHAP to create a SHAP-explainer model. We then run test data through the SHAP-explainer model to generate a SHAP contribution value for each input feature. We run this experiment with a 10-fold cross-validation setup and calculate the average SHAP contribution value. The average contribution value of each feature, for all features and models, was recorded (due to space limitations, only one summary of these is shown here, as a stacked bar chart in Figure 6).

### 5.2. Domain Differences

To understand the differences between source and target domains, we use KS tests to quantify the difference for each feature. The KS test can be used to compare two samples without making an assumption about the distribution of data. The null hypothesis is that the two samples, source and target data, come from the same distribution. The KS test produces a KS-statistic and *p*-value. The KS-statistic represents the maximum distance between the source data and the target data distributions. The *p*-value represents the significance level, e.g., less than 0.05. We used the maximum distance between the source data and the target data distribution curves (KS-statistic) to provide insights into the differences in features between these two domains.

### 5.3. Domain Adaptation

Domain adaptation aims to transform the source data distribution to be similar to the target data distribution. The intention is to use latent features constructed using source data to complement the target data. We propose the following approach to adapt the feature distribution of the source data to mimic the feature distribution of target data. For each feature, the adaptation steps are:

1. Group the source data and the target data in *N* quantiles, where *N* should be selected to ensure that we have sufficient data for each quantile, e.g., more than 50 samples. In this study, we selected *N* = 10, after experimenting with various *N* values.
2. For each corresponding source and target quantiles, we calculate *scale* and then adapt/adjust the source feature values:
  (27) $$\begin{array}{r} \text{scale}=\frac{\left(\text{max}\left(\text{target\_value}\right)-\text{min}\left(\text{target\_value}\right)\right)}{\left(\text{max}\left(\text{source\_value}\right)-\text{min}\left(\text{source\_value}\right)\right)} \end{array}$$
  (28) $$\begin{array}{r} \text{offset}=\left(\text{source\_value}-\text{min}\left(\text{source\_value}\right)\right)*\text{scale} \end{array}$$
  (29) $$\begin{array}{r} \text{adapted\_source\_value}=\text{min}\left(\text{target\_value}\right)+\text{offset} \end{array}$$

The adapted source feature values are used to initially train the neural network before the last layers are retrained using the target features.

Based on observations of explainer models and feature differences, we adapted different sets of features before training, and then trained and tested using the method described in Section 4 on transfer learning. We then compared the performance of models (using AUC) with different adaptation sets, and transferred models without adaptation. We found that adapting all features significantly reduces accuracy, so we tried different combinations of features to adapt to find the most accurate adapted models.

### 5.4. Experiments and Results

#### 5.4.1. Transfer Learning and Explainability

Table 3 shows the AUC comparison for the target and the transferred models. The accuracy of the transferred models was better than the target models; AUC improved by 0.042 or 7% for the MD domain, and 0.0224 or 3.6% for the SB domain, respectively. This is in line with the results of Suryanto et al. (2019).

**Table 3:** Target model vs. transferred model.

**Domain**	**Experiment**	**AUC**	**Improvement**	***p*-value**
CD to MD	Training using Target only	0.5971(0.0823)		
CD to MD	Training using Source then retraining the last layer using Target	0.6391(0.0856)	0.0420 (7.0%)	<0.01
CD to SB	Training using Target only	0.6194(0.0456)		
CD to SB	Training using Source then retraining the last layer using Target	0.6419(0.0509)	0.0224 (3.6%)	<0.01

More interestingly, Figure 6 shows a comparison of feature contributions from source, target, and transferred models. For the MD (and SB, not shown) domains, “cover” becomes the top contributing feature of the more accurate transferred models. However, it was the least contributing feature for both the target and source models for CD to MD transfer and a weak contributor in the SB target model. All other features contributed less in the transferred model than in the target model, no matter how much they contributed in the source model for CD to MD transfer. It was similar for CD to SB transfer except for interest rate, which was one of the top contributors in the source model (CD domain). These explainer models indicate that transfer learning improves accuracy by boosting the contribution of weak features in the target domain.

To understand the contribution of “cover,” we investigated the difference in the value distribution for “cover” between source and target domains. Comparing the difference in value distribution between source and target with KS statistics we observed that source CD compared to target MD has a larger (and significant) difference than between source CD and target SB. This difference is larger for small values of cover (< 20) in both distribution comparisons but is more obvious for the CD to MD source–target difference where the source distribution has over twice as many loans as the target for the smallest value of cover. This difference is much less for the CD to SB source–target difference, particularly for larger values of cover. Further results are presented in the following section.

#### 5.4.2. Domain Difference

Table 4 lists the KS statistics for CD vs. MD as well as CD vs. SB. It shows that some features were very different between source and target domains but some were similar. It also shows that different pairings of source and target domains had different patterns in feature differences. For example, “cover” was very different between CD and MD with a KS-statistic of 0.2729, but similar between CD and SB with a KS-statistic of 0.0502.

**Table 4:** Kolmogorov-Smirnov (KS) of input features.

		**CD vs. MD**		**CD vs. SB**	
**No**	**Short name**	**KS stats**	**KS** ***p*****-value**	**KS stats**	**KS** ***p*****-value**
1	term_36m	0.0407	<0.24	0.0357	<0.20
2	term_60m	0.0407	<0.24	0.0357	<0.20
3	grade_n	0.0792	<0.01	0.0984	<0.01
4	sub_grade_n	0.0884	<0.01	0.1069	<0.01
5	int_rate_n	0.0941	<0.01	0.1033	<0.01
6	revol_util_n	**0.2248**	<0.01	**0.2292**	<0.01
7	emp_length_n	0.0242	<0.85	0.0749	<0.01
8	dti_n	**0.1502**	<0.01	**0.2295**	<0.01
9	installment_n	**0.3005**	<0.01	0.0671	<0.01
10	annual_inc_n	0.0670	<0.01	0.0906	<0.01
11	loan_amnt_n	**0.2899**	<0.01	0.0813	<0.01
12	cover	**0.2736**	<0.01	0.0585	<0.01

*Values in bold represent large differences that are statistically significant*.

#### 5.4.3. Domain Adaptation

To further understand the contribution of cover, we applied our proposed domain adaptation function. Before we trained the transfer model on the source data, we adapted the cover on the source data to make it similar to the target data, and then applied the transfer learning technique to produce an “adapted” and transferred model. The AUC tests for these adapted and transferred models are listed in Table 5 where they are compared to the transferred model without adaptation. We ran paired *t*-tests to test the “improvements” (AUC increase or decrease) shown in Tables 5–**7**; all improvements were statistically significant with *p* < 0.01. Since this data was normally distributed *t*-tests were appropriate. Adapting cover works for CD to MD transfer with an AUC 0.01 (1.6%) higher than the transfer-only model, but AUC decreases for a CD to SB transfer.

**Table 5:** Adapted model vs. transferred model.

**Domain**	**Experiment**	**AUC (s.d.)**	**Improvement**	***p*-value**
CD to MD	Transfer only	0.6391(0.0856)		
CD to MD	Transfer with cover adapted	0.6491(0.0824)	0.0100 (1.6%)	<0.01
CD to SB	Transfer only	0.6419(0.0509)		
CD to SB	Transfer with cover adapted	0.6361(0.0502)	–0.0058 (–0.9%)	<0.01

We also examined SHAP changes with the improved model. After adapting cover, it became the fourth highest contributing feature, and the top three contributing features (int_rate_n, term_36m, and annual_inc_n) were the top contributing features of the target and source models.

We tested various permutations of features to adapt to find the most accurate model for the CD to MD transfer and to establish an optimal strategy for seeking the most accurate adapted model. The experiments on the CD to MD transfer are listed in Table 6. When adapting all features, or adapting credit grade and related features, model accuracy was significantly reduced, with AUC 0.1771 (27.7%) or 0.1756 (27.5%) lower than the transfer-only model. Adapting only features with a high KS-statistic (over 0.15), i.e., revolving utility, debt to income ratio, installment, loan amount, and cover, improved accuracy with AUC 0.0172 (2.7%) higher than the transfer-only model. Adding related features, i.e., annual income (annual_inc_n)—which is used to derive cover (a high KS feature), further improved accuracy, with AUC 0.0209 (3.3%) higher than the transfer-only model. Removing credit history features that are intrinsic to the borrower, i.e., revolving utility and debt to income ratio, produced an even more accurate model, with AUC 0.0257 (4.0%) higher than the transfer-only model.

**Table 6:** Adapted model vs. transferred model in CD to MD transfer.

**Experiment**	**AUC (s.d.)**	**Improvement**	***p*-value**
Transfer only	0.6391(0.0856)		
Adapt all features	0.4620(0.3048)	–0.1771 (–27.7%)	<0.01
Adapt credit grade and related features, i.e., grade, sub-grade, interest rate	0.4635(0.3052)	–0.1756 (–27.5%)	<0.01
Adapt features with high KS, i.e., revolving utility, debt to income ratio, installment, loan amount and cover	0.6563(0.0806)	0.0172 (2.7%)	<0.01
Adapt features with high KS and related features, i.e., revolving utility, debt to income ratio, installment, loan amount, cover and annual income	0.6600(0.07417)	0.0209 (3.3%)	<0.01
Adapt features with high KS and related features less credit history features, i.e., installment, loan amount, cover and annual income	0.6649(0.0731)	0.0257 (4.0%)	<0.01

Grade, sub-grade, revolving utility (revol_util_n), and debt to income ratio (dti_n) are features derived from credit history, which are intrinsic to the borrower and are usually highly correlated with the lending outcome, i.e., default or not. The interest rate in the lending club data is derived directly from grade and sub-grade, so we consider it as a credit history feature in our experiment.

We use a similar strategy for the CD to SB transfer. The AUC comparison with the transfer-only model is shown in Table 7. Adapting all features, or credit grade related features, significantly reduced model accuracy, with AUC 0.123 (19.3%) or 0.1106 (17.2%) lower than the transfer-only model, respectively. We tested adaptation of the features that we adapted for the most accurate model for the CD to MD transfer, which has a low KS-statistic from CD and SB comparisons. This adapted model was slightly less accurate, with an AUC 0.0015 (0.2%) lower than the transfer-only model. Adapting features with a high KS-statistic, i.e., revolving utility and debt to income ratio, improved model accuracy slightly, with AUC 0.0018 (0.3%) higher than the transfer-only model. These two features do not have related features, and both were credit history features, so we could not improve accuracy further as we did with the CD to MD transfer.

**Table 7:** Adapted model vs. transferred model in CD to SB transfer.

**Experiment**	**AUC (s.d.)**	**Improvement**	***p*-value**
Transfer only	0.6419(0.0509)		
Adapt all features	0.5189(0.1666)	–0.123 (–19.2%)	<0.01
Adapt credit grade and related features, i.e., grade, sub-grade, interest rate	0.5313(0.1624)	–0.1106 (–17.2%)	<0.01
Adapt features used in CD to MD transfer, i.e., installment, loan amount, cover, annual income	0.6404(0.0475)	–0.0015 (–0.2%)	<0.01
Adapt features with high KS, i.e., revolving utility and debt to income ratio	0.6437(0.0495)	0.0018 (0.3%)	<0.01

Additionally, we investigated the explainability of the most accurate models using SHAP to assess the feature contributions of the most accurate adapted models compared to the source and target models. Through domain adaptation, the contribution of weak features increased in the most accurate adapted models. For the CD to MD transfer, the contribution of annual income, cover, installment, and loan amount increased. For the CD to SB transfer, the contribution of annual income, term 36 months or 60 months, cover, employment length, installment, and loan amount increased.

To evaluate the effectiveness of our adaptation approach, we compared KS values before and after adaptation for the most accurate models, as shown in Table 8. The reduction in KS-statistics was between 44.8 and 90.3%, and for features, with high KS-statistics (over 0.15) the reductions were all above 67.4%. Our adaptation approach successfully reduced the differences between the distribution of the source data and the target data.

**Table 8:** Kolmogorov-Smirnov test to compare source data and target data, before and after the source data is adapted, ACD is the abbreviation for Adapted Credit card and Debt consolidation data.

**Feature**	**Without adaptation**			**With adaptation**			**Reduction**
	**Domain**	**KS-stats**	***p*-value**	**Domain**	**KS-stats**	***p*-value**	
installment	CD to MD	0.3005	<0.01	ACD to MD	0.0293	<0.64	90.3%
annual_inc	CD to MD	0.0670	<0.01	ACD to MD	0.0369	<0.34	44.8%
loan_amnt	CD to MD	0.2899	<0.01	ACD to MD	0.0681	<0.01	76.5%
cover	CD to MD	0.2736	<0.01	ACD to MD	0.0892	<0.01	67.4%
revol_util	CD to SB	0.2292	<0.01	ACD to SB	0.0536	<0.01	76.6%
dti	CD to SB	0.2295	<0.01	ACD to SB	0.0301	<0.39	86.9%

## 6. Discussion

Transfer learning improves model accuracy by generating intermediate features from the source domain to be selected for retraining on the target domain. This intermediate-feature-generation concept is similar to “self taught learning” proposed by Raina et al. (2007), which constructed higher-level features using unlabeled data, except that in this article, we used labeled data from the source domain.

The contribution of a weak feature from the target domain increased because it was complemented by new intermediate features from the source domain. We tested an adaptation approach taking the outcome label into consideration. But this did not improve model accuracy. The reason was that the population of positive (outcome=1) cases was too small in the already small target dataset.

Adapting strong credit history features, such as grade and sub-grade, significantly reduced model accuracy, while removing features related to credit history from the adaptation list improved model accuracy. Adapting credit history related features *without consideration of the outcome label* generates unrealistic instances, e.g., changing a borrower's credit grade from high to low without adjusting the outcome from not default to default. These unrealistic instances can negatively impact model accuracy.

## 7. Conclusion and Future Study

In this article, we have proposed and evaluated a method of gradually shifting the contribution from the source to the target domain during transfer learning. The *PSC* method in a structured way varies network architecture and hyperparameters to find the best balance to learn from source and target domains. While in this article, certain design choices were made manually, the overarching aim was to create a framework within which such changes could be optimized automatically, in our setting to find the best Gini score.

In terms of interpretability of models for credit assessment, we found that SHAP is an effective tool in explaining why transfer learning improves the accuracy of credit scoring models. In our experiments transfer learning lifted the contribution of weak features, thereby improving overall prediction accuracy.

Domain adaptation with the right set of features further improved the accuracy of transfer learning models. However, adapting all features normally reduces model accuracy significantly. Reasons to select features to adapt include: differences in feature distribution between source and target domain, quantified by KS-statistics; relationships to already selected features; and domain specific knowledge, e.g., the credit history features intrinsic to the borrowers.

Through domain adaptation, the contribution of weaker features increased in the most accurate adapted models. An adaptation approach that significantly reduces KS-statistics has been critical in producing a successful domain adaptation algorithm.

If a model constructed by a machine learning approach may affect capital requirements, then such models need to be reviewed by a regulatory institution. However, business cases where our approach is applicable are in many domains that do not fall in these categories.

For future study, the proposed strategy to select features for domain adaptation produces more accurate credit scoring models, but the execution of the strategy requires human intervention in observing and applying domain knowledge. We will further explore methods to automate this selection strategy, so it can be a pre-processing step for fully automated transfer learning. Although in this article we have focused on transfer learning based on deep networks, other model architectures could be used. For example, Goussies et al. (2014) and Segev et al. (2016) have used ensembles of tree learners as the basis for transfer learning. Comparison of deep networks with such approaches could be investigated as part of further study.

The use of alternatives to KS statistics to estimate the distance between distributions, such as KL-divergence, should be investigated. SHAP is an indirect method to understand the impact of latent intermediate features. A further study exploring and explaining latent intermediate features could improve our understanding of transfer learning and domain adaptation, and better meet transparency and compliance requirements.

Finally, we note that although the significant improvements in accuracy demonstrated are small in terms of percentage improvements, such improvements in real world lending could be of substantial economic importance in reducing lenders' losses due to loan defaults.

## Data Availability Statement

The software and instructions for downloading and pre-processing the data are provided at the following link to help Reproducible Research URL: https://gitlab.com/research-study/ecmlpkdd2020.

## Author Contributions

HS, CG, and AG contributed to conception and design of the study, wrote the first draft of the manuscript. HS contributed to implementations and statistical analysis. HS, AM, and MB contributed to research and analysis and wrote sections of the manuscript. All authors contributed to manuscript revision, read, and approved the submitted version.

## Funding

This study received funding from Rich Data Corporation, Sydney, Australia. The funder was not involved in the study design, collection, analysis, interpretation of data, the writing of this article or the decision to submit it for publication.

## Conflict of Interest

HS, AM, CG, and AG are employed by Rich Data Corporation, Sydney, Australia. The remaining author declares that the research was conducted in the absence of any commercial or financial relationships that could be construed as a potential conflict of interest.

## Publisher's Note

All claims expressed in this article are solely those of the authors and do not necessarily represent those of their affiliated organizations, or those of the publisher, the editors and the reviewers. Any product that may be evaluated in this article, or claim that may be made by its manufacturer, is not guaranteed or endorsed by the publisher.

## Footnotes

^1^Available from https://www.lendingclub.com/info/download-data.action

^2^The hyper parameters optimization has been done before this step.

^3^See https://github.com/slundberg/shap
